# Clinical Characteristics of Carbapenem-Resistant Gram-Negative Bloodstream Infections and Fungemia Among High-Risk Pediatric Patients Receiving Empirical Antifungal Therapy

**DOI:** 10.3390/pathogens15070714

**Published:** 2026-07-07

**Authors:** Asuman Akar

**Affiliations:** Department of Pediatrics, Division of Pediatric Infectious Diseases, Dicle University Faculty of Medicine, Diyarbakır 21280, Turkey; asuman.akar@dicle.edu.tr

**Keywords:** healthcare-associated infections, carbapenem-resistant Gram-negative bacteria, fungemia, pediatric infections, central venous catheter, antimicrobial resistance, mortality

## Abstract

Background: Healthcare-associated bloodstream infections remain a significant cause of morbidity and mortality in hospitalized children, particularly in intensive care settings. Carbapenem-resistant Gram-negative bacterial (CR-GNB) bloodstream infections and fungemia may present with overlapping clinical features. This can complicate empirical treatment decisions in resource-limited settings. This study evaluated baseline clinical and laboratory characteristics associated with CR-GNB bloodstream infections and fungemia among high-risk pediatric patients. Methods: This retrospective observational cohort study included pediatric patients aged 0–18 years who were evaluated at the time of clinical deterioration and blood culture collection for suspected healthcare-associated bloodstream infection before empirical antifungal therapy initiation for the index episode. Patients who subsequently received empirical antifungal therapy between May 2023 and September 2025 were retrospectively screened. Of the 240 screened patients, 103 met the inclusion criteria and were classified into CR-GNB (n = 56) and fungemia (n = 47) groups based on blood culture results. Clinical, laboratory, and microbiological data were analyzed using univariate and multivariable statistical methods. Results: Observed 90-day all-cause mortality was higher in the CR-GNB group than in the fungemia group (50.0% vs. 29.8%, *p* = 0.038). Central venous catheter use was more frequent (91.1% vs. 48.9%, *p* = 0.006), and platelet counts were lower (median: 120 × 10^9^/L vs. 259 × 10^9^/L, *p* = 0.011) in patients with CR-GNB bloodstream infections. In multivariable analysis, thrombocytopenia (OR: 4.22, 95% CI: 1.35–13.17; *p* = 0.013) and central venous catheter use (OR: 5.53, 95%: CI 1.89–16.26; *p* = 0.002) were independently associated with CR-GNB bloodstream infections. The model showed moderate discrimination (AUC = 0.786). Conclusion: In this selected high-risk cohort, thrombocytopenia and central venous catheter use were associated with CR-GNB bloodstream infections. Observed mortality was higher in the CR-GNB group, but this finding should be interpreted with caution as adjusted mortality analysis and standardized severity assessment were not performed. These findings are hypothesis-generating and require validation in larger prospective studies before guiding empirical treatment decisions.

## 1. Introduction

Healthcare-associated infections (HAIs) remain a major cause of morbidity and mortality in pediatric patients, particularly in intensive care settings, where they contribute to prolonged hospitalization and increased healthcare burden [1,2]. The reported incidence of HAIs in hospitalized children ranges from 4.0% to 13.0% depending on the patient population and healthcare setting [3,4]. Several risk factors have been associated with pediatric HAIs, including prolonged hospitalization, central venous and urinary catheter use, mechanical ventilation, broad-spectrum antibiotic exposure, severe sepsis, neutropenia, and total parenteral nutrition [5,6,7,8,9,10].

Bacteria (both Gram-positive and -negative) and fungi are among the most common pathogens causing pediatric HAIs [7]. In recent years, antimicrobial resistance among Gram-negative pathogens has emerged as a major global health challenge, particularly due to the increased prevalence of carbapenem-resistant Gram-negative bacteria (CR-GNB) [11,12,13,14,15,16]. Mortality rates associated with CR-GNB bloodstream infections may exceed 40.0%, especially in critically ill patients and in intensive care settings [15,17,18,19,20,21]. The limited availability of effective antimicrobial treatment options further complicates the management of these infections [9,19,20,22].

Fungal bloodstream infections also represent a major cause of morbidity and mortality in hospitalized and immunocompromised pediatric patients. Commonly reported risk factors include hematologic malignancies, transplantation, prolonged neutropenia, central venous catheter use, prolonged intensive care unit stay, and prior broad-spectrum antibiotic exposure [23,24,25,26]. Reported mortality rates in invasive fungal infections vary widely and may reach 20.0–70.0% in high-risk pediatric populations [27,28].

In clinical practice, distinguishing CR-GNB bloodstream infections from fungemia may be particularly challenging in critically ill pediatric patients because both conditions frequently share overlapping clinical and epidemiological characteristics, including prolonged hospitalization, exposure to invasive devices, elevated inflammatory markers, intensive care admission, and previous broad-spectrum antimicrobial use [7,14]. This diagnostic uncertainty may complicate empirical treatment decisions, particularly in resource-limited healthcare settings where rapid molecular diagnostics and advanced microbiological testing are not routinely available.

Despite the clinical importance of this issue, pediatric studies directly comparing CR-GNB bloodstream infections and fungemia remain limited. Therefore, this study aimed to evaluate baseline clinical and laboratory characteristics associated with CR-GNB bloodstream infections and fungemia among high-risk pediatric patients assessed for suspected healthcare-associated bloodstream infection before empirical antifungal therapy initiation.

## 2. Materials and Methods

### 2.1. Study Design and Setting

This retrospective observational cohort study was conducted at a tertiary-care university hospital serving a socioeconomically disadvantaged population in southeastern Türkiye. The primary objective was to evaluate baseline clinical and laboratory characteristics associated with CR-GNB bloodstream infections and fungemia among high-risk pediatric patients at the time of suspected bloodstream infection. The secondary objective was to compare observed 90-day all-cause mortality between the two groups, defined as all-cause mortality occurring within 90 days after blood culture collection.

Ninety-day all-cause mortality was selected because shorter endpoints, such as 14-day or 30-day mortality, may underestimate the clinical burden of CR-GNB bloodstream infections and fungemia in critically ill pediatric patients with prolonged intensive care stays and late-onset complications. This time window is consistent with previous outcome studies of CR-GNB bloodstream infections [15,29,30]. All-cause mortality was preferred over infection-attributable mortality to reduce misclassification bias inherent to the retrospective design.

Because rapid molecular diagnostics, carbapenemase characterization, fungal biomarkers, and antifungal susceptibility testing were not routinely available at our institution during the study period, empirical antimicrobial decision-making primarily relied on clinical assessment and conventional microbiological methods.

### 2.2. Study Population

Pediatric patients aged 0–18 years who received empirical antifungal therapy for a suspected healthcare-associated bloodstream infection between 1 May 2023, and 1 September 2025, were retrospectively screened. All patients were identified at the time of clinical deterioration, when healthcare-associated bloodstream infection was suspected, and blood cultures were obtained. At this time point, none of the patients had received empirical antifungal therapy for the index infectious episode. Empirical antifungal treatment was initiated only after blood culture collection and clinical assessment by pediatric infectious diseases specialists. Therefore, this study evaluates baseline clinical and laboratory findings obtained before empirical antifungal therapy.

At our institution, empirical antifungal therapy is commonly initiated in critically ill pediatric patients with prolonged hospitalization, exposure to invasive devices, persistent fever despite broad-spectrum antimicrobial therapy, or clinical deterioration suggestive of invasive infection. Therefore, this cohort represents a high-risk population for whom differentiating resistant bacterial bloodstream infections from fungemia may be clinically challenging.

Bloodstream infections were confirmed microbiologically, and only patients with positive blood cultures were included in the study. Positive cultures obtained from non-blood clinical specimens were excluded. Patients with Gram-positive bacterial growth, patients with concomitant bacterial and fungal bloodstream infections (mixed bloodstream infections), duplicate isolates, or missing clinical data were also excluded. Of the 240 patients screened, only microbiologically confirmed bloodstream infections fulfilled all eligibility criteria and were included in the final analysis (n = 103).

Eligible patients were categorized into two groups according to blood culture results:

(1) Carbapenem-resistant Gram-negative bloodstream infections (CR-GNB group) and
(2) the fungemia group.

Institutional protocol for empirical antifungal and antimicrobial therapy: At our institution, empirical antifungal therapy is initiated in critically ill pediatric patients who have experienced prolonged hospitalization, invasive device exposure, persistent fever despite at least 72 h of broad-spectrum antimicrobial therapy, or clinical deterioration suggestive of invasive fungal infection. First-line empirical antifungal therapy consists primarily of fluconazole for hemodynamically stable patients without recent azole exposure and liposomal amphotericin B for hemodynamically unstable patients or those with prior azole exposure. Empirical broad-spectrum antibacterial therapy, usually piperacillin–tazobactam, meropenem, or cefepime, was administered according to institutional sepsis practice and consultation with pediatric infectious diseases. The type, timing, and duration of empirical therapy were recorded but were not included in the outcome analysis because treatment approaches were heterogeneous.

### 2.3. Data Collection

Clinical, demographic, microbiological, and laboratory data were obtained from electronic medical records. Recorded variables included age, sex, admission unit, underlying diseases, intensive care unit stay, invasive device use, prior antimicrobial exposure, mechanical ventilation, total parenteral nutrition, and mortality.

Thrombocytopenia was defined as a platelet count <150 × 10^9^/L, and neutropenia was defined as an absolute neutrophil count <500 × 10^9^/L according to institutional pediatric reference standards.

Clinical and laboratory variables were recorded at the time of clinical deterioration, and blood culture collection was conducted for suspected bloodstream infection. This occurred prior to empirical antifungal therapy initiation for the index episode. Thus, all laboratory parameters analyzed in this study represent pretreatment baseline values.

### 2.4. Microbiological Procedures

Blood cultures were obtained aseptically from peripheral venous blood samples at the time of suspected bloodstream infection and were processed using the BACT/ALERT system (bioMérieux, Marcy-l’Étoile, France).

Only bloodstream isolates recovered from blood culture samples were included in the analysis. Bacterial species-level identification was performed using conventional microbiological methods, including Gram staining, colony morphology, oxidase testing, and biochemical profiling with API 20E and API 20NE identification systems (bioMérieux, Marcy-l’Étoile, France) as needed. Fungal species-level identification was performed using colony morphology on Sabouraud dextrose agar, microscopic examination, germ tube testing for presumptive Candida albicans identification, and chromogenic agar for preliminary differentiation of Candida species. Confirmatory biochemical testing was performed using the VITEK 2 Compact system (bioMérieux, France). Matrix-assisted laser desorption/ionization time-of-flight mass spectrometry was not available during the study period; therefore, definitive identification of some non-albicans Candida species and rare non-fermentative Gram-negative isolates may have been limited.

Antimicrobial susceptibility testing was performed using routine microbiological laboratory methods, including the Kirby–Bauer disk diffusion method, using EUCAST criteria applicable during the study period.

Carbapenem resistance was defined as resistance to at least one carbapenem agent (meropenem or imipenem) based on phenotypic susceptibility testing results. CR-GNB isolates included *Klebsiella pneumoniae*, *Acinetobacter* spp., and *Pseudomonas aeruginosa*.

Fungal bloodstream infections were defined by positive blood cultures yielding *Candida species*. Due to institutional resource limitations, carbapenemase characterization and antifungal susceptibility testing could not be routinely performed.

### 2.5. Statistical Analysis

Data were analyzed using SPSS version 24.0 (IBM Corp., Chicago, IL, USA). The normality of continuous variables was assessed using the Shapiro–Wilk test and visual inspection of histograms. Continuous variables were expressed as the mean ± standard deviation or median with interquartile range, depending on the distribution. Categorical variables were summarized as frequencies and percentages.

Comparisons between the CR-GNB bloodstream infection group and the fungemia group were performed using Student’s *t*-test or Mann–Whitney U test for continuous variables, as appropriate. Categorical variables were compared using the Pearson chi-square test or Fisher’s exact test when expected cell counts were small.

To identify factors associated with CR-GNB bloodstream infection, binary logistic regression analysis was performed using infection group as the dependent variable. Variables with clinical relevance and/or a *p*-value < 0.10 in univariate analyses were considered for inclusion in the multivariable model. To reduce the risk of overfitting due to the limited sample size, the final model was restricted to a limited number of clinically relevant variables. Variables were selected for the final multivariable model based on their clinical relevance, biological plausibility, and their association with CR-GNB bloodstream infection in univariate analyses, while avoiding inclusion of highly overlapping variables because of the limited sample size. Model calibration was not formally assessed because the model was exploratory, the sample size was modest, and the number of outcome events was limited. Therefore, the model was interpreted only as an association model rather than a validated predictive tool.

Odds ratios (ORs) with 95% confidence intervals (CIs) were reported. Model discrimination was assessed using receiver operating characteristic (ROC) curve analysis and the area under the curve (AUC). The regression model was only used to evaluate factors associated with CR-GNB bloodstream infection and was not interpreted as a model predicting mortality.

Ninety-day mortality was compared descriptively between groups using univariate analysis. No separate multivariable mortality model was constructed due to the limited sample size and number of outcome events. Therefore, mortality findings were interpreted as observed between-group differences rather than independent mortality predictors.

A two-sided *p*-value < 0.05 was considered statistically significant.

## 3. Results

A total of 103 pediatric patients with microbiologically confirmed bloodstream infections were included. Of these, 63 (61.2%) were male. The mean age was 4.1 ± 4.8 years (median: 2 years). Most patients were admitted to intensive care units, mainly the pediatric intensive care unit (n = 45, 43.7%) and the pediatric surgical intensive care unit (n = 16, 15.5%). Overall, 90-day mortality was 40.8% (42/103).

The most common underlying condition was acute leukemia (n = 27, 26.2%), followed by gastrointestinal malformations (n = 23, 22.3%), immunodeficiency (n = 14, 13.6%), cystic fibrosis or chronic lung disease (n = 12, 11.6%), ventriculoperitoneal shunt (n = 10, 9.7%), and burns (n = 5, 4.8%). Mechanical ventilation (70.9%), urinary catheterization (38.8%), and total parenteral nutrition (20.4%) were required in 73, 40, and 21 patients, respectively.

CR-GNB bloodstream infections were identified in 56 patients (54.4%). The most common CR-GNB pathogen was *Klebsiella pneumoniae* (n = 29, 51.7%), followed by *Acinetobacter* spp. (n = 20, 35.7%) and *Pseudomonas aeruginosa* (n = 7, 12.5%).

Fungemia was detected in 47 patients (45.6%). *Candida parapsilosis* was the most frequently isolated fungal pathogen (n = 24, 51.1%), followed by *Candida albicans* (n = 17, 36.2%) and *Candida tropicalis* (n = 6, 12.8%). The demographic, clinical, and microbiological characteristics of the cohort are summarized in Table 1.

Patients were categorized into two groups according to microbiological findings: the CR-GNB bloodstream infection group and the fungemia group.

Platelet counts were lower in the CR-GNB group than in the fungemia group (median: 120 × 10^9^/L vs. 259 × 10^9^/L, *p* = 0.011). No between-group differences were detected in white blood cell count, absolute neutrophil count, hemoglobin, C-reactive protein, the duration of prior piperacillin–tazobactam/meropenem/cefepime exposure, or the length of intensive care unit stay.

Detailed comparisons of laboratory and clinical parameters between the two groups are presented in Table 2.

Clinical characteristics and outcomes were compared between the CR-GNB bloodstream infection and fungemia groups. Observed 90-day mortality was higher in the CR-GNB group than in the fungemia group (50.0% vs. 29.8%, *p* = 0.038).

Central venous catheter use was more frequent among patients with CR-GNB bloodstream infections (91.1% vs. 48.9%, *p* = 0.006). Prior exposure to piperacillin–tazobactam/meropenem/cefepime and thrombocytopenia were also more common in the CR-GNB group (*p* = 0.045 and *p* = 0.008, respectively).

No between-group differences were observed in total parenteral nutrition, mechanical ventilation, or urinary catheterization.

Detailed comparisons of clinical characteristics between the two groups are presented in Table 3. Because of the retrospective design and heterogeneity of treatment approaches, antimicrobial and antifungal treatment regimens were not analyzed comparatively.

A multivariable logistic regression analysis was performed to evaluate factors associated with CR-GNB bloodstream infections. The platelet count was converted into a dichotomous thrombocytopenia variable and defined as <150 × 10^9^/L for multivariable analysis.

Thrombocytopenia (OR: 4.22, 95% CI: 1.35–13.17; *p* = 0.013) and central venous catheter use (OR: 5.53, 95% CI: 1.89–16.26; *p* = 0.002) were independently associated with CR-GNB bloodstream infections. Other evaluated variables, including total parenteral nutrition, mechanical ventilation, duration of prior broad-spectrum antibacterial exposure, and intensive care unit stay, were not independently associated with CR-GNB bloodstream infections.

**Figure 1:**
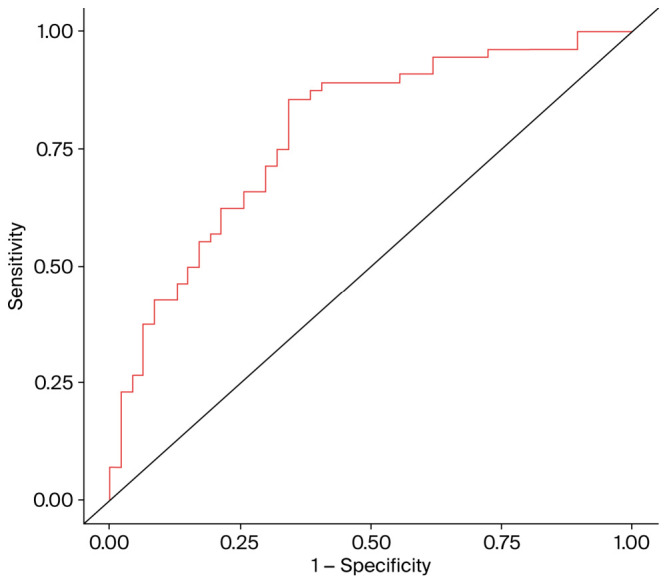
Receiver operating characteristic (ROC) curve of the multivariate logistic regression model (AUC = 0.786).

The detailed results of the multivariable analysis are presented in Table 4. The regression model yielded an AUC of 0.786 (Figure 1). Based on the classification table, the model demonstrated a positive predictive value of 72.9% and a negative predictive value of 70.5%, as well as an accuracy of 71.8%, sensitivity of 76.8%, and specificity of 66.0%. Accordingly, the regression model was interpreted as an exploratory association model rather than a validated diagnostic prediction model. These performance measures are summarized in Table 5.

## 4. Discussion

The principal findings of this study were that thrombocytopenia and central venous catheter use were independently associated with CR-GNB bloodstream infections among high-risk pediatric patients evaluated before empirical antifungal therapy initiation. The 90-day all-cause mortality observed was also higher in the CR-GNB group than in the fungemia group.

The overall mortality rate in our cohort was consistent with previously reported mortality rates in healthcare-associated bloodstream infections and critically ill pediatric populations [29,30,31,32,33,34]. The higher observed mortality in the CR-GNB group may reflect the substantial clinical burden associated with antimicrobial resistance, severe underlying illness, and limited therapeutic options in resistant Gram-negative infections [5,6,16,29,30,34]. Similar findings have been reported in previous studies of critically ill patients with CR-GNB bloodstream infections, particularly in intensive care settings [29,30,33,34]. In contrast, mortality associated with fungemia may vary based on immunosuppression, underlying disease, and fungal pathogen [35,36].

Importantly, the observed difference in mortality should be interpreted in the context of unmeasured baseline illness severity. Standardized pediatric severity scores, such as PRISM III, PELOD-2, and PIM-2, were not consistently documented in this retrospective dataset [37,38]. Therefore, residual confounding by indication cannot be excluded, and the lower observed mortality in the fungemia group may partly reflect baseline differences in illness severity rather than the infecting pathogen alone.

In the present study, thrombocytopenia and central venous catheter use were independently associated with CR-GNB bloodstream infections. These findings are consistent with previous reports identifying invasive device exposure and hematological abnormalities as markers of severe healthcare-associated infections [21,25,26,39,40,41,42,43,44,45]. Although CVC use has often been discussed in relation to candidemia, prolonged exposure to invasive devices may also facilitate colonization by multidrug-resistant Gram-negative organisms, particularly in critically ill patients receiving broad-spectrum antimicrobial therapy [21,25,26]. Thrombocytopenia may reflect systemic inflammation, sepsis-related platelet consumption, antimicrobial exposure, or pathogen-specific virulence factors in CR-GNB bloodstream infections [21,39,40]. Other evaluated variables, including neutropenia, mechanical ventilation, total parenteral nutrition, and intensive care unit stay, were not independently associated with CR-GNB bloodstream infections in the multivariable analysis.

The pathogen distribution observed in our study was generally consistent with previous reports. *Klebsiella pneumoniae* was the most common CR-GNB isolate, whereas *Candida parapsilosis* was the predominant fungal pathogen [46,47,48]. However, antifungal susceptibility testing and carbapenemase characterization could not be performed routinely due to institutional resource limitations.

Previous studies have identified prolonged hospitalization, intensive care unit stay, invasive device exposure, prior broad-spectrum antimicrobial use, and immunosuppression as major risk factors for both CR-GNB bloodstream infections and fungemia in pediatric patients [9,16,49,50,51,52,53,54,55,56]. In our cohort, these variables were evaluated when available. Among them, central venous catheter use and thrombocytopenia remained independently associated with CR-GNB bloodstream infections.

A clinically relevant observation in this study was the considerable overlap between baseline characteristics of the CR-GNB and fungemia groups. Examples include exposure to intensive care, invasive devices, and previous use of broad-spectrum antimicrobial therapy. This overlap may complicate empirical treatment decisions in settings where rapid molecular diagnostics and fungal biomarkers are unavailable [19,20].

Although thrombocytopenia and central venous catheter use remained independently associated with CR-GNB bloodstream infections, the clinical applicability of this model should be interpreted cautiously. The overlap between CR-GNB bloodstream infections and fungemia, the relatively small sample size, and the wide confidence intervals limit its immediate use for clinical decision-making. Therefore, these findings should be considered hypothesis-generating and require external validation in larger prospective multicenter studies before implementation in routine clinical practice. Importantly, thrombocytopenia and central venous catheter use should not be used in isolation to guide empirical antimicrobial therapy. These variables should be interpreted only within the overall clinical context, together with the patient’s underlying disease, severity of illness, prior antimicrobial exposure, local resistance patterns, and microbiological data when available.

Overall, our findings indicate that thrombocytopenia and central venous catheter use were associated with CR-GNB bloodstream infections in a selected high-risk pediatric cohort. However, several clinical characteristics overlapped with the fungemia group. These observations may improve clinical awareness in resource-limited settings, but they should not be interpreted as a validated diagnostic tool.

## 5. Conclusions

In this retrospective observational cohort of high-risk pediatric patients evaluated before empirical antifungal therapy, thrombocytopenia and central venous catheter use were associated with CR-GNB bloodstream infections. Observed 90-day all-cause mortality was higher in the CR-GNB group; however, this finding should be interpreted cautiously because adjusted mortality analysis and standardized severity assessment were not performed. These findings are hypothesis-generating and should be validated in larger prospective studies before being used to guide empirical treatment decisions.

## 6. Limitations of the Study

This study has several limitations. First, it was conducted at a single center with a relatively small sample size, which may limit the generalizability of the findings and reduce the statistical power for some secondary analyses. Second, the study was performed in a resource-limited setting where advanced diagnostic methods—including molecular sepsis panels, carbapenemase characterization, and antifungal susceptibility testing—were not routinely available. Therefore, microbiological characterization and resistance profiling could not be fully evaluated; classification of carbapenem resistance relied on phenotypic susceptibility testing results.

Third, the retrospective study design may have introduced selection bias. Although clinical and laboratory variables were obtained before empirical antifungal therapy initiation for the index episode, the study population comprised patients who subsequently received empirical antifungal treatment due to high clinical suspicion for invasive fungal infection. Therefore, this cohort likely represents a subgroup of critically ill patients with higher baseline severity and increased suspicion for invasive infection, and the findings should not be interpreted as a comparison of the untreated natural course of CR-GNB bloodstream infections and fungemia.

In addition, several variables that may influence mortality and clinical outcomes, including standardized illness severity scores, source of bloodstream infection, timing and adequacy of antimicrobial therapy, catheter removal, interventions for source control, and detailed microbiological characterization, were not consistently available due to the retrospective design. Therefore, observed mortality differences between groups may have been influenced by baseline disease severity and treatment-related factors rather than pathogen group alone.

## Figures and Tables

**Table 1:** Baseline demographic and clinical characteristics according to infection group.

Characteristic	CR-GNB (n = 56)	Fungemia (n = 47)
**Sex**		
Female	25 (44.6%)	15 (31.9%)
Male	31 (55.4%)	32 (68.1%)
**Admission unit**		
Pediatric intensive care unit	22 (39.3%)	23 (48.9%)
General pediatric ward	7 (12.5%)	9 (19.1%)
Pediatric surgical intensive care unit	11 (19.6%)	5 (10.6%)
Neurosurgery intensive care unit	0 (0.0%)	2 (4.3%)
Pediatric hematology–oncology unit	11 (19.6%)	4 (8.5%)
Neonatal intensive care unit	3 (5.4%)	1 (2.1%)
Plastic surgery intensive care unit	2 (3.6%)	3 (6.4%)
**Underlying disease**		
Immunodeficiency	7 (12.5%)	7 (14.9%)
Chronic lung disease/cystic fibrosis	5 (8.9%)	7 (14.9%)
Gastrointestinal malformation	14 (25.0%)	9 (19.1%)
Ventriculoperitoneal shunt/cerebral palsy sequelae	3 (5.4%)	7 (14.9%)
Malignancy	21 (37.5%)	6 (12.8%)
Sepsis without underlying disease	5 (8.9%)	8 (17.0%)
Burn	1 (1.8%)	3 (6.4%)
***Microbiological findings***		
Carbapenem-resistant Gram-negative bacteria	56 (54.4)	
– *Klebsiella pneumoniae*	29 (51.7)	
– *Acinetobacter* spp.	20 (35.7)	
– *Pseudomonas aeruginosa*	7 (12.5)	
Fungal bloodstream infections	47 (45.6)	
– *Candida parapsilosis*	24 (51.1)	
– *Candida albicans*	17 (36.2)	
*– Candida tropicalis*	6 (12.8)	

**Table 2:** Comparison of laboratory and clinical parameters between the CR-GNB bloodstream infection and fungemia groups.

Variable	CR-GNB (Median, IQR)	Fungemia (Median, IQR)	*p*-Value
White blood cell count	8450 (4665–14,025)	9300 (6800–15,080)	0.31
Neutrophil count	4310 (1328–8600)	5040 (2825–8250)	0.61
Hemoglobin (g/dL)	10 (9–11)	10 (9–12)	0.25
Platelet count (×10^9^/L)	120 (37–312.7)	259 (108–406.5)	0.011
CRP (mg/L)	60.5 (16–201)	51 (13–213)	0.27
Prior piperacillin–tazobactam/meropenem/cefepime exposure (days)	21 (14–31.5)	19 (11–33)	0.76
ICU stay (days)	34.5 (10.8–86)	25 (12–116)	0.44

**Table 3:** Comparison of clinical characteristics between the CR-GNB bloodstream infection and fungemia groups.

Variable	CR-GNB (n = 56)	Fungemia (n = 47)	*p*-Value
90-day all-cause mortality	28 (50.0%)	14 (29.8%)	0.038
Total parenteral nutrition	15 (26.8%)	6 (12.8%)	0.079
Central venous catheter	51 (91.1%)	23 (48.9%)	0.006
Mechanical ventilation	41 (73.2%)	32 (68.1%)	0.568
Urinary catheter	20 (35.7%)	20 (42.6%)	0.478
Prior piperacillin–tazobactam/meropenem/cefepime exposure	55 (98.2%)	41 (87.2%)	0.045
Thrombocytopenia	30 (53.6%)	13 (27.7%)	0.008

**Table 4:** Multivariable logistic regression analysis of factors associated with CR-GNB bloodstream infections.

Variable	β (Estimate)	Wald	*p*-Value	OR	95% CI
Mechanical ventilation	0.20	0.34	0.731	1.22	0.39–3.86
Total parenteral nutrition	0.79	1.64	0.199	2.21	0.66–7.42
Central venous catheter	1.71	9.69	0.002	5.53	1.89–16.26
Absolute neutrophil count	0.26	1.36	0.175	1.00	−2.11–4.46
Thrombocytopenia	1.44	6.18	0.013	4.22	1.35–13.17
ICU stay (days)	−0.001	0.35	0.558	0.99	0.99–1.00
Prior broad-spectrum antibacterial exposure (days)	−0.02	1.92	0.166	0.97	0.94–1.01

**Table 5:** Predictive performance of the multivariable logistic regression model.

Metric	Value
Accuracy	71.8%
Sensitivity	76.8%
Specificity	66.0%
Positive predictive value (PPV)	72.9%
Negative predictive value (NPV)	70.5%
AUC	0.786

Note. PPV, positive predictive value; NPV, negative predictive value; AUC, area under the receiver operating characteristic curve.

## Data Availability

The data that support the findings of this study are available on request from the corresponding author. The data are not publicly available due to privacy and ethical restrictions.

## Abbreviations

The following abbreviations are used in this manuscript:

HAIs	Healthcare-associated infections
CR-GNB	carbapenem-resistant Gram-negative bacteria
AMR	antimicrobial resistance
CVC	central venous catheter
